# Incarceration as a determinant of health: bioethical and legal dilemmas in the care of pregnant women

**DOI:** 10.1590/1806-9282.20260642

**Published:** 2026-08-10

**Authors:** Fábio Roberto Cabar

**Affiliations:** 1 Universidade de São Paulo, Hospital das Clínicas, Faculty of Medicine, Division of Obstetrics – São Paulo (SP), Brazil.

The practice of medicine within carceral settings is, by definition, a challenge to the professional’s technical and ethical integrity. However, when the “patient-inmate” dyad shifts to that of the “pregnant-incarcerated woman,” the complexity transcends clinical practice and enters a territory where bioethics and law are dramatically strained. Physicians often serve as the sole mediators between human dignity and the punitive rigor of the State^1^.

## BIOETHICAL CONFLICT: THE “DOUBLE VULNERABILITY”

While principalist bioethics provides a framework for navigation, in the carceral setting, the compass often fails^2^. The incarcerated pregnant woman personifies a superimposed vulnerability: the restriction of autonomy inherent to the prison system and the biological and social dependence characteristic of pregnancy.

Non-maleficence and the “Innocent Third Party”: The principle of non-maleficence extends to the fetus. The prison environment—characterized by insalubrity, overcrowding, and chronic stress—functions as a “social teratogen.” Forcing a newborn to begin life in an environment of deprivation constitutes an extension of punishment to an individual who has committed no crime, violating the legal and ethical precept that punishment must not transcend the person of the convict^3,4^.

Justice and Equity: The principle of equity, a pillar of the Brazilian Unified Health System, is severely compromised. Access to diagnostic imaging, nutritional supplementation, and high-risk obstetric monitoring is frequently hindered by bureaucracy or neglect, creating an unacceptable disparity between the free and the incarcerated pregnant woman^5^.

## DIMINISHED AUTONOMY AND THE ETHICS OF CARE

In the field of clinical bioethics, autonomy presupposes the individual’s freedom to decide on interventions concerning their own body. In prison, this autonomy is mitigated by disciplinary power. The pregnant patient often finds herself in a position of subordination, where consent for medical procedures may be influenced by the power dynamics of custody^2,6^.

It is the physician’s duty to ensure that decisions regarding the mode of delivery, postpartum contraception, and breastfeeding are made freely and with informed consent. The practice of shackling women during labor, although legally prohibited in several jurisdictions (including Brazil), represents the pinnacle of autonomy and dignity violations, constituting institutional violence that the physician is ethically obligated to prevent^1,7^.

## IMPACTS ON NEONATAL DEVELOPMENT: TOXIC STRESS

Medical literature is unequivocal: the prison environment is incompatible with healthy neonatal development. The chronic toxic stress experienced by the incarcerated pregnant woman elevates maternal cortisol levels, which correlates with intrauterine growth restriction and prematurity^8^.

In the postpartum period, the right to breastfeeding and skin-to-skin contact is frequently interrupted by administrative decisions regarding inmate transfers. The disruption of the primary affective bond for purely punitive reasons ignores the permanent neurobiological repercussions for the child and the deleterious effects on the mother’s mental health^9^.

## THE PHYSICIAN’S ROLE WITHIN THE LEGAL FRAMEWORK

In Brazil, Collective Habeas Corpus 143.641/SP and Law 13.769/2018 establish the substitution of preventive detention with house arrest for pregnant women and mothers^10,11^. In this context, the physician acts as a “witness of reality.” Our clinical assessments are not merely medical records; they are fundamental documents that allow the judiciary to understand the incompatibility between neonatal survival and the carceral environment. By documenting the insalubrity of the unit or the lack of specialized care, the professional provides the necessary technical foundation for the protection of life^1,11^.

## CONCLUSION

Healthcare for incarcerated pregnant women demands a stance of “medical advocacy.” This is not a debate over the patient’s culpability, but rather a safeguard to ensure that a judicial sentence does not transition into institutionalized medical neglect. The physician must recognize themselves as the guarantor of the autonomy and health of the mother–child dyad, ensuring that childbirth and the beginning of life do not occur under the shadow of state abandonment. Medical ethics compels us to be the voice for humanization where the system sees only retribution.

## Data Availability

The datasets generated and/or analyzed during the current study are available from the corresponding author upon reasonable request.

## References

[1] Federal Council of Medicine (2019). Code of medical ethics: CFM resolution No. 2,217, of September 27, 2018.

[2] Beauchamp TL, Childress JF (1994). Principles of biomedical ethics.

[3] Ventura M, Simas L, Larouze B (2015). Management of pregnancy in prison: a challenge for public health and human rights. Rev Saúde Pública.

[4] Presidency of the Republic (Brazil) (1988). Constitution of the Federative Republic of Brazil of 1988.

[5] Official Gazette of the Union (Brazil). Law No. 8,080, of September 19, 1990 (1990). Provides for conditions for the promotion, protection, and recovery of health.

[6] United Nations (2010). The Bangkok rules: United Nations rules for the treatment of women prisoners.

[7] Official Gazette of the Union (Brazil). Law No. 13,434, of April 12, 2017 (2017). Amends the code of criminal procedure to ensure that handcuffs are not used on pregnant women during labor.

[8] Shonkoff JP, Garner AS (2012). The lifelong effects of early childhood adversity and toxic stress. Pediatrics.

[9] Official Gazette of the Union (Brazil). Law No. 13,257, of March 8, 2016 (2016). Provides for public policies for early childhood.

[10] Supreme Federal Court (Brazil). Collective habeas corpus 143,641/SP (2018). Rapporteur: Justice Ricardo Lewandowski.

[11] Official Gazette of the Union (Brazil). Law No. 13,769, of December 19, 2018 (2018). Amends the code of criminal procedure and the penal execution law to establish the substitution of preventive prison with house arrest for pregnant women.

